# Luminal concentrations of L- and D-lactate in the rectum may relate to severity of disease and outcome in septic patients

**DOI:** 10.1186/cc5102

**Published:** 2006-11-20

**Authors:** Vibeke L Jørgensen, Nanna Reiter, Anders Perner

**Affiliations:** 1 Department of Anaesthesia and Intensive Care, Herlev Hospital, Herlev Ringvej 75, DK-2730 Herlev, Denmark; 2 Department of Intensive Care, Rigshospitalet, University of Copenhagen, Blegdamsvej, DK-2100 Copenhagen Ø, Denmark

## Abstract

**Introduction:**

Little is known about the condition of the large bowel in patients with sepsis. We have previously demonstrated increased concentrations of L-lactate in the rectal lumen in patients with abdominal septic shock. The present study was undertaken to assess the concentrations of L- and D-lactate in rectal lumen and plasma in septic patients including the possible relation to site of infection, severity of disease, and outcome.

**Methods:**

An intensive care unit observational study was conducted at two university hospitals, and 23 septic patients and 11 healthy subjects were enrolled. Participants were subjected to rectal equilibrium dialysis, and concentrations of L- and D-lactate in dialysates and plasma were analysed by spectrophotometry.

**Results:**

Luminal concentrations of L-lactate in rectum were related to the sequential organ failure assessment scores (*R*^2 ^= 0.27, *P *= 0.01) and were higher in non-survivors compared to survivors and healthy subjects (mean [range] 5.0 [0.9 to 11.8] versus 2.2 [0.4 to 4.9] and 0.5 [0 to 1.6] mmol/l, respectively, *P *< 0.0001), with a positive linear trend (*R*^2 ^= 0.53, *P *< 0.0001). Also, luminal concentrations of D-lactate were increased in non-survivors compared to survivors and healthy subjects (1.1 [0.3 to 2.5] versus 0.3 [0 to 1.2] and 0.1 [0 to 0.8] mmol/l, respectively, *P *= 0.01), with a positive linear trend (*R*^2 ^= 0.14, *P *= 0.04). Luminal concentrations of L- and D-lactate were unaffected by the site of infection. Plasma concentrations of L-lactate were also increased in non-survivors compared to survivors (3.8 [1.7 to 7.0] versus 1.5 [0 to 3.6] mmol/l, *P *< 0.01). In contrast, plasma concentrations of D-lactate were equally raised in non-survivors (0.4 [0.1 to 0.7] mmol/l) and survivors (0.3 [0.1 to 0.6] mmol/l) compared with healthy subjects (0.03 [0 to 0.13] mmol/l).

**Conclusion:**

In patients with severe sepsis and septic shock, luminal concentrations of L- and D-lactate in the rectum were related to severity of disease and outcome.

## Introduction

Intestinal failure may contribute to morbidity and mortality in sepsis [1]. However, little is known about the condition of the large bowel in these patients. It is likely that metabolic products, including L-lactate, do escape the intestines, but most of it may be metabolised by the liver [2,3], hampering systemic assessment. This raises the possibility that increased lactate production in the intestine goes undetected when measuring systemic values.

Luminal equilibrium dialysis is a valid, non-invasive method for the estimation of extra-cellular concentrations of small molecules (<12 kDa) in rectal mucosa [4]. When full equilibrium is obtained, the concentration in the dialysate will reflect the average extra-cellular concentration on the epithelium covered by the membrane during the time of equilibration. Using this method, we have previously demonstrated increased concentrations of L-lactate in the rectal lumen in patients with septic shock and abdominal focus of infection [5] and in patients undergoing cardiopulmonary bypass [6]. More importantly, we have shown that luminal concentrations of L-lactate relate to colorectal permeability in patients with severe sepsis [7], indicating pathophysiological relevance. In patients, it is unknown whether luminal concentrations of lactate reflect mucosal values or whether they are affected by systemic lactate. In animals, however, studies using the microdialysis technique, in which the probes are much smaller, have shown that luminally measured lactate is the better marker of occlusive ischaemia and is unaffected by hyperlactataemia [8]. Others have proposed the plasma values of the D-isoform of lactate, which is a metabolic product of luminal bacteria, as a possible marker of intestinal perfusion disturbances in critically ill patients [9,10].

To advance our understanding of these potential markers of intestinal metabolism in sepsis, several questions have to be answered, including both the correlation between them and their relation to clinical parameters. Therefore, the present study was undertaken to assess concentrations of L- and D-lactate in the rectal lumen and systemic circulation in septic patients and the possible relation to site of infection, severity of disease, and outcome.

## Materials and methods

The regional ethics committee of Copenhagen County, Denmark, approved the study protocol, and informed written consent was obtained from the closest relative prior to study.

### Patients

In the period of 2002 to 2004, patients with severe sepsis or septic shock, as defined by consensus criteria [11], were included if the condition had persisted for more than 24 hours in spite of source control, including any surgery. During the study, the treating clinician decided patient management, and all patients were mechanically ventilated and had been fluid-resuscitated prior to study by using repeated boluses until the mean arterial blood pressure or the dose of noradrenaline was stable. Fluid balance was maintained with normal saline during the study. Patients who fulfilled one of the following criteria were not evaluated for inclusion: (a) treatment with inotropic or vasopressor drugs other than noradrenaline, (b) pathology of the rectum or sigmoid colon, (c) any changes in therapy in the hour prior to study, (d) systemic hypoxia (PaO_2 _[partial pressure of arterial oxygen] <8 kPa) or severe hypercapnia (PaCO_2 _[partial pressure of arterial carbon dioxide] >7 kPa), (e) gastrointestinal bleeding, or (f) need of haemodialysis or haemofiltration during the study period of four hours. Healthy subjects were enrolled among hospital staff after informed written consent, and all were free of medication for at least one month prior to study.

### Protocol

After enrolment in the study, participants were subjected to rectal equilibrium dialysis, and clinical variables were registered at baseline and again after four hours. Simplified acute physiology score (SAPS) II was calculated based on values of the first 24 hours after admission, and sequential organ failure assessment (SOFA) score was calculated from values of the preceding 24 hours.

### Concentrations of L- and D-lactate in rectal lumen and plasma

The concentrations of L- and D-lactate in the rectal lumen were assessed by equilibrium dialysis as previously described [5]. In brief, a semi-permeable bag of cellulose (cutoff value of 12 kDa; Sigma-Aldrich, St. Louis, MO, USA) containing 4 ml of 10% Dextran 40 in isotonic saline (Meda AB, Solna, Sweden) was placed in the rectal lumen for four hours, which is the estimated time required for 100% equilibrium for lactate *in vivo *[4]. Blood was sampled from the arterial line, and dialysate and plasma concentrations of L- and D-lactate were measured by spectrophotometry using stereo-specific lactate dehydrogenase as previously described [12]. In five patients (three non-survivors and two survivors), plasma was not sampled.

### Statistical analysis

Data are presented as mean values with ranges. Prior to analysis, Bartlett's test for equal variance was used, and if significant differences were observed, the data were log_10_-transformed. Data were compared by one-way analysis of variance and post-test for linear trend or by unpaired or paired Student's *t *test. Relationships between variables were assessed by linear regression analysis, and goodness of fit was evaluated by residual plots and visual inspection. All calculations were performed using GraphPad Prism 4.1 (GraphPad Software, Inc., San Diego, CA, USA), and *P *values less than 0.05 (two-tailed) were considered significant.

## Results

Sixteen patients with septic shock and seven patients with severe sepsis were included of which 11 (48%) had died 28 days after study. Descriptive statistics are shown in Table 1, and selected clinical variables during study are shown in Table 2.

### Luminal and plasma concentrations of L-lactate

**Figure 1:**
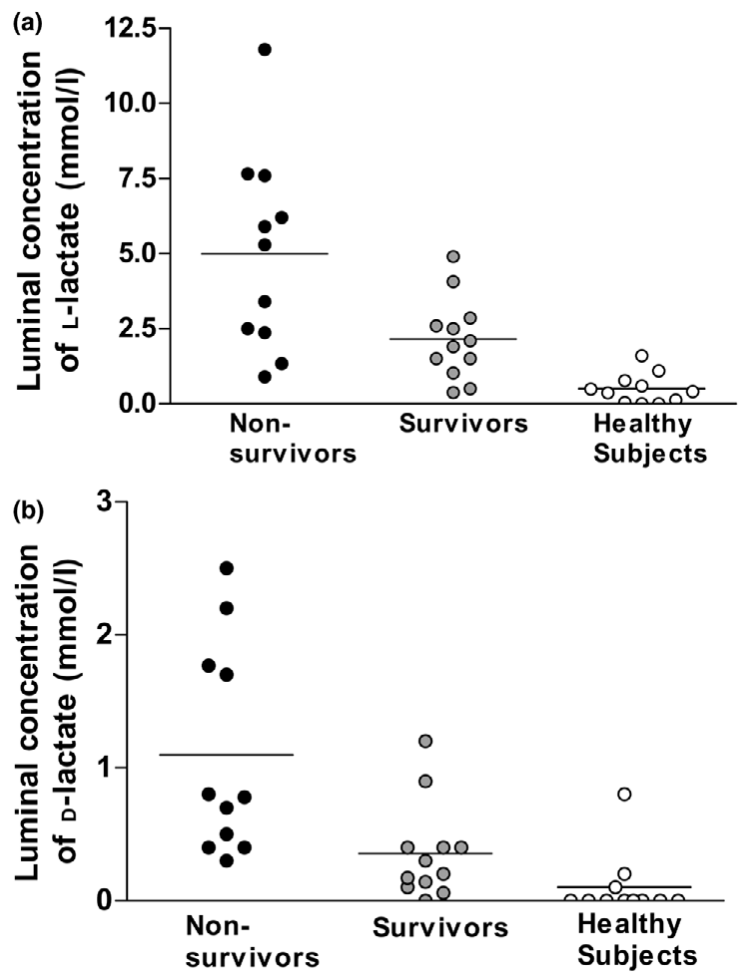
Luminal concentrations of L-lactate (a) and D-lactate (b) in non-surviving and surviving septic patients and healthy subjects. Dots represent values of individual patients, and bars represent mean values, which were significantly (*P *< 0.0001 [a] or *P *= 0.01 [b]) different by one-way analysis of variance (after log_10 _transformation) with a positive linear trend (*P *< 0.0001 [a] or *P *= 0.04 [b]).

**Figure 2:**
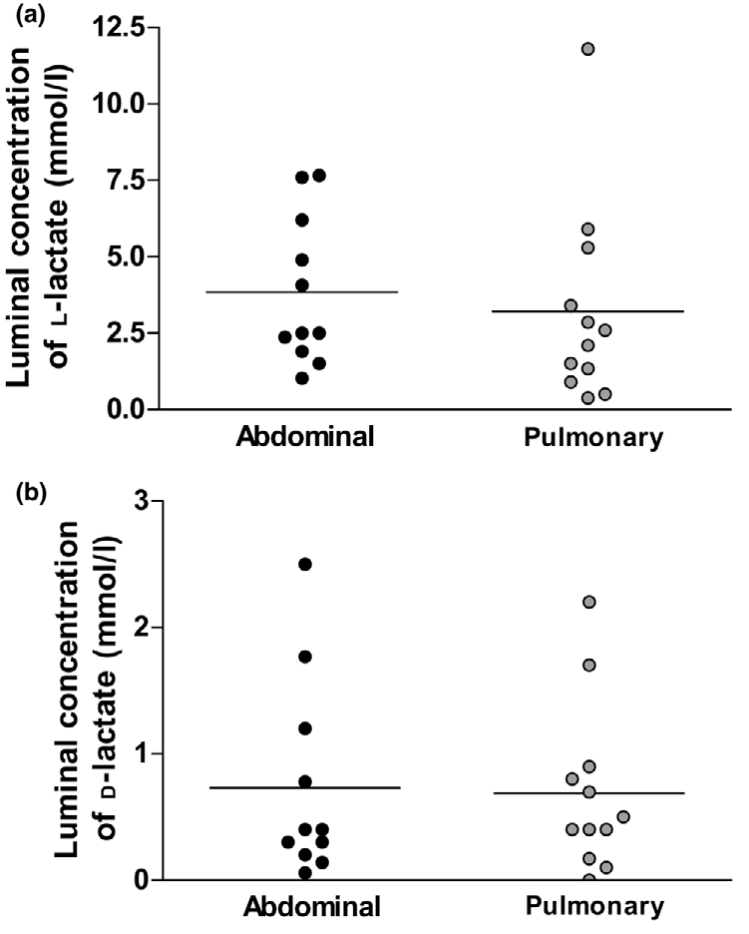
Luminal concentrations of L-lactate (a) and D-lactate (b) in septic patients differentiated by focus of infection. Dots represent values of individual patients, and bars represent mean values, which were not different by unpaired *t *test (*P *= 0.61 [a] or *P *= 0.89 [b]).

Rectal luminal concentrations of L-lactate were increased in non-survivors compared with survivors and healthy subjects (5.0 [0.9 to 11.8] versus 2.2 [0.4 to 4.9] and 0.5 [0 to 1.6] mmol/l, respectively, *P *< 0.0001; Table 3; Figure 1a), with a positive linear trend (*R*^2 ^= 0.53, *p *< 0.0001). Luminal L-lactate concentrations did not differ between patients with 'abdominal' and 'pulmonary' sepsis (Figure 2a) but were positively related to SOFA scores (*R*^2 ^= 0.27, *P *= 0.01) and arterial concentrations of L- and D-lactate (*R*^2 ^= 0.23, *P *= 0.04 and *R*^2 ^= 0.21, *P *= 0.05, respectively). In contrast, luminal concentrations of L-lactate were unrelated to SAPS II (*P *= 0.09) and dose of noradrenaline (*n *= 16, *P *= 0.07). Luminal L-lactate values were higher than plasma values in 11 of 18 patients (luminal-arterial gradient 0.7 [-3.4 to 4.7] mmol/l, *P *= 0.20, mean versus 0 by one-sample *t *test, *n *= 18), but the gradient did not differ between non-survivors and survivors (Table 3). Six of the 11 patients also had a positive luminal-arterial gradient of D-lactate, but the two gradients were not related (*P *= 0.37). Plasma concentrations of L-lactate were stable during study (Table 2) and were increased in the group of non-survivors compared with survivors (3.8 [1.7 to 7.0] versus 1.5 [0 to 3.6] mmol/l; Table 3).

### Luminal and plasma concentrations of D-lactate

The concentrations of D-lactate in the rectal lumen were also increased in non-surviving patients compared with survivors and healthy subjects (1.1 [0.3 to 2.5] versus 0.4 [0 to 1.2] and 0.1 [0 to 0.8] mmol/l, respectively, *P *= 0.01; Table 3; Figure 1b), with a positive linear trend (*R*^2 ^= 0.14, *P *= 0.04). Luminal concentrations of D-lactate did not differ between patients with 'abdominal' and 'pulmonary' sepsis (Figure 2b) and were unrelated to luminal concentrations of L-lactate (*P *= 0.16) and plasma concentrations of D-lactate (*P *= 0.93). Luminal D-lactate values were higher than plasma values in 13 of 18 patients (luminal-arterial gradient 0.4 [-0.3 to 2.2] mmol/l, *P *= 0.02, mean versus 0 by one-sample *t *test, *n *= 18), but the gradient did not differ between non-survivors and survivors (Table 3). Plasma concentrations of D-lactate were significantly increased in non-surviving and surviving patients when compared with healthy subjects (0.4 [0.1 to 0.7] and 0.3 [0.1 to 0.6] versus 0.03 [0 to 0.13] mmol/l, respectively, *P *< 0.01; Table 3), but there was no difference between non-survivors and survivors (*P *= 0.22).

## Discussion

We observed increased concentrations of L- and D-lactate in the rectal lumen in septic patients, which were independent of the site of infection. More importantly, these changes correlated to severity of disease and outcome. This indicates that elevated luminal concentrations of L- and D-lactate are markers of metabolic dysfunction in the large bowel. There was also a weak positive relationship between luminal and plasma concentrations of L-lactate, which may cast doubt on the usefulness of the regional marker. Our study was not designed to identify independent predictors of mortality or to assess the clinical usefulness of luminal equilibrium dialysis. This may only be addressed in a larger study, but it may be difficult to statistically differentiate the effect of luminal L-lactate on outcome from the effect of hyperlactataemia, because the latter is an independent predictor of mortality [13-17]. Luminal L-lactate values were not related to SAPS II or noradrenaline dose, which is likely to be a type 2 error due to the low number of patients. The concept of the luminal-arterial gradient was evolved for gastric partial pressure of carbon dioxide (PCO_2_) to express mucosal perfusion based on the assumption that an increased veno-arterial PCO_2 _gradient reflects tissue hypoperfusion [18]. The present data suggest that the information to gain from the lactate gradients is that the rectum becomes a producer of L- and D-lactate in sepsis.

The plasma concentrations of D-lactate observed in the present study are within the range observed by others [9,10,19,20]. In contrast to our study, Poeze and colleagues [9] found increased plasma concentrations in non-surviving septic patients compared with survivors. The reason for this discrepancy cannot be assessed, but they only included patients within 24 hours of shock debut, whereas we only included patients after 24 hours. Given that plasma D-lactate was correlated to gastric PCO_2 _in their study, it may be that low intestinal perfusion early in sepsis is associated with bad outcome. In a more recent study, however, the same group showed that gastric PCO_2 _was unrelated to outcome in early shock [21]. These discrepancies currently cannot be explained. The novelty of the present study is the set of additional measurements of luminal values of L- and D-lactate. The much higher luminal concentration of L-lactate compared with D-lactate indicates that the mucosa is the source of most of the lactate produced. D-lactate is produced by bacteria as an intermediate in the formation of short-chain fatty acids [19]. Some *Lactobacilli *express a DL-lactate racemase, which may convert the isomeric forms in a concentration-dependent process [22]. Thus, increased luminal L-lactate may result in increased luminal D-lactate and subsequently elevated plasma values, because D-lactate is not readily metabolised in the liver. Plasma D-lactate may therefore be a marker of L-lactate production in the large bowel, but our data suggest that it is less sensitive than luminally measured L- or D-lactate, both of which discriminated survivors from non-survivors. Alternatively, increased rate of fermentation by the colonic flora can cause D-lactic acidosis as seen in patients with short bowel syndrome [22]. In these patients, increased input of carbohydrates into the colonic lumen enhances the fermentation process. In septic patients, it may be speculated that altered colonic flora due to antibiotics also could contribute. In any case, the large bowel may suffer from several potential hits in sepsis, including altered perfusion, nutrients, microbial flora, and inflammation, and all of these may have contributed to our observations.

Lactate has for 70 years been considered a marker of anaerobic glycolysis, and clinical practice to optimise oxygen delivery in septic patients has evolved around this concept. In recent years, the understanding of lactate formation and metabolism has been challenged and extended. Controversy exists whether increased lactate represents hypoxia or aerobic glycolysis [23]. The study by Rivers and colleagues [24] in patients with severe sepsis and hyperlactataemia demonstrated that early goal-directed therapy targeting markers of flow was associated with a more rapid decrease in lactate levels and improved outcome. Similarly, Levy and colleagues [25] have shown that the lactate-to-pyruvate ratio in plasma was markedly elevated in patients with septic shock, suggesting a hypoxic origin of hyperlactataemia in these patients. On the other hand, non-hypoxic causes of hyperlactatemia can be observed. Studies of raised systemic lactate in human endotoxaemia and sepsis indicate that the adrenergic surge contributes through increased muscle Na^+^K^+^ATPase activity and glycolysis [26,27]. In contrast, the source of lactate in the intestines is unknown, and extrapolating data from other tissues is not straightforward, because the mucosa contains many different cell types. The metabolism of the epithelium differs from all other tissue as short-chain fatty acids are nutrients in epithelial cells in the large bowel, making glucose-dependent mechanisms of increased lactate production unlikely, at least in these cells. Even though decreased lactate clearance in the liver may contribute to elevated systemic values in septic patients, this is unlikely to explain our observation of elevated concentrations of lactate in the intestinal lumen.

Previous studies of markers of metabolism in the gut in septic patients have used tonometry to assess PCO_2 _in the gastric lumen. Very little is known about differences in barrier dysfunction between different parts of the gastrointestinal tract. In animal studies, the large bowel has been observed to be more susceptible to endotoxaemia than the small bowel [28]. Moreover, toxic production from the rectal lumen may get direct access to the systemic circulation via the iliac veins. Because luminal L-lactate may correlate to colorectal permeability [7], there is a theoretical rationale to assess L-lactate in this part of the gut. Future studies may establish the role for the measurement of L-lactate in the rectal lumen in septic patients, in whom dynamic assessment during treatment may be possible [29].

## Conclusion

Luminal concentrations of L- and D-lactate in the rectum are increased in septic patients and may relate to severity of disease and outcome. Further studies may indicate whether altered perfusion, nutrients, microbial flora, inflammation, or aerobic glycolysis contributes to these observations.

## Key messages

• This study reports for the first time systemic and luminal concentrations of both lactate enantiomers in septic patients.

• Markedly increased concentrations of L- and D-lactate were observed in the rectal lumen in patients with severe sepsis and septic shock, independent of the site of infection.

• More importantly, these changes correlated to severity of disease and outcome, indicating pathophysiological relevance.

## Abbreviations

PCO_2 _= partial pressure of carbon dioxide; SAPS = simplified acute physiology score; SOFA = sequential organ failure assessment.

## Competing interests

The authors declare that they have no competing interests.

## Authors' contributions

VLJ and AP were involved in design, data collection and analysis, drafting of manuscript, and revisions. NR was involved in data collection and analysis. All authors read and approved the final manuscript.

## Figures and Tables

**Table 1:** Characteristics of participants

	Non-survivors^a ^(*n *= 11)	Survivors^a ^(*n *= 12)	Healthy subjects (*n *= 11)
Age (years)	65 (53 to 79)^b^	60 (26 to 79)^b^	25 (23 to 31)
Male/female	6/5	9/3	5/6
Laparotomy	4	5	N/A
Colonic surgery (ascending)	1	1	N/A
Site of infection (pulmonary/abdominal)	6/5	6/6	N/A
Isolated bacteria (Gram-negative/Gram-positive/none)	5/5/1	6/6/0	N/A
SAPS^c ^II	49 (14 to 93)	56 (33 to 98)	N/A
Severe sepsis/septic shock	2/9	5/7	N/A
SOFA^d ^score	15 (7 to 23)^e^	11 (4 to 17)	N/A

Values are given as numbers or means (ranges). ^a^Survival analysis based on data at day 28 after study. ^b^*P *< 0.001 compared with healthy subjects. ^c^Simplified acute physiology score (admission values). ^d^Sequential organ failure assessment (in the preceding 24 hours). ^e^*P *< 0.01 compared with survivors. N/A, not applicable.

**Table 2:** Clinical variables for non-surviving and surviving septic patients at baseline and after four hours

	Non-survivors^a ^(*n *= 11)		Survivors^a ^(*n *= 12)	
	Baseline	Four hours	Baseline	Four hours

Heart rate (beats per minute)	96 (59 to 121)	92 (65 to 126)	100 (63 to 121)	100 (68 to 120)
Mean arterial pressure (mm Hg)	75 (62 to 90)^b^	76 (64 to 97)	87 (71 to 108)	83 (56 to 103)
Noradrenaline dose^c ^(μg/kg per minute)	0.20 (0.06 to 0.44)	0.24 (0.02 to 0.60)	0.37 (0.04 to 1.7)	0.37 (0.04 to 1.6)
Plasma L-lactate^d ^(mmol/l)	2.4 (1 to 3.8)	2.7 (1.1 to 6.2)	1.5 (0.5 to 3.6)	1.5 (0.6 to 2.9)
Arterial pH	7.35 (7.28 to 7.48)	7.32 (7.22 to 7.38)	7.33 (7.20 to 7.48)	7.34 (7.21 to 7.46)

Values are given as means (ranges). ^a^Survival analysis based on data at day 28 after study. ^b^*P *< 0.05 compared with baseline values in survivors. ^c^Nine patients in the non-surviving group and seven in the surviving group received noradrenaline. ^d^Values from ABL blood gas autoanalyser (Radiometer, Copenhagen, Denamrk).

**Table 3:** Concentrations of L- and D-lactate in rectal lumen and plasma in septic patients and healthy subjects

	Non-survivors^a ^(*n *= 11)	Survivors^a ^(*n *= 12)	Healthy subjects (*n *= 11)
Luminal L-lactate (mmol/l)	5.0^b ^(0.9 to 11.8)	2.2 (0.4 to 4.9)	0.5 (0 to 1.6)
Plasma L-lactate (mmol/l)	3.8^c, d ^(1.7 to 7.0)	1.6^e ^(0.5 to 3.6)	1.8 (0.8 to 3.1)
Δ-L-lactate, lumen – plasma (mmol/l)	0.8 (-3.4 to 4.7)	0.5 (-1.1 to 3.5)	-1.5^f ^(-2.5 to -0.3)
Luminal D-lactate (mmol/l)	1.1^g ^(0.3 to 2.5)	0.4 (0 to 1.2)	0.1 (0 to 0.8)
Plasma D-lactate (mmol/l)	0.4^c, h ^(0.1 to 0.7)	0.3^e, h ^(0 to 0.6)	0.03 (0 to 0.13)
Δ-D-lactate, lumen – plasma (mmol/l)	0.8 (-0.3 to 2.2)	0.1 (-0.3 to 0.7)	0.01 (-0.13 to 0.2)

Values are given as means (ranges). ^a^Survival analysis is based on data from day 28 after study. ^b^*P *< 0.0001 by one-way analysis of variance (ANOVA) after log_10 _transformation with positive linear trend, *P *< 0.0001. ^c^Only values from eight patients are given (see Materials and methods). ^d^*P *< 0.01 compared with survivors by unpaired *t *test. ^e^Only values from 10 patients are given (see Materials and methods). ^f^Value different from 0 by one-sample *t *test, *P *= 0.02. ^g^*P *= 0.01 by one-way ANOVA with positive linear trend *P *= 0.04. ^h^*P *< 0.01 versus healthy subjects by unpaired *t *test.
